# Supplementary material for: Live imaging of wound angiogenesis reveals macrophage orchestrated vessel sprouting and regression
Source: EMBO J. 2018 Jun 4;37(13):e97786. doi: 10.15252/embj.201797786 (PMC6028026; doi:10.15252/embj.201797786)
Supplement: Supplementary file 1 — Expanded View Figures PDF [file EMBJ-37-e97786-s001.pdf]

Expanded View Figures

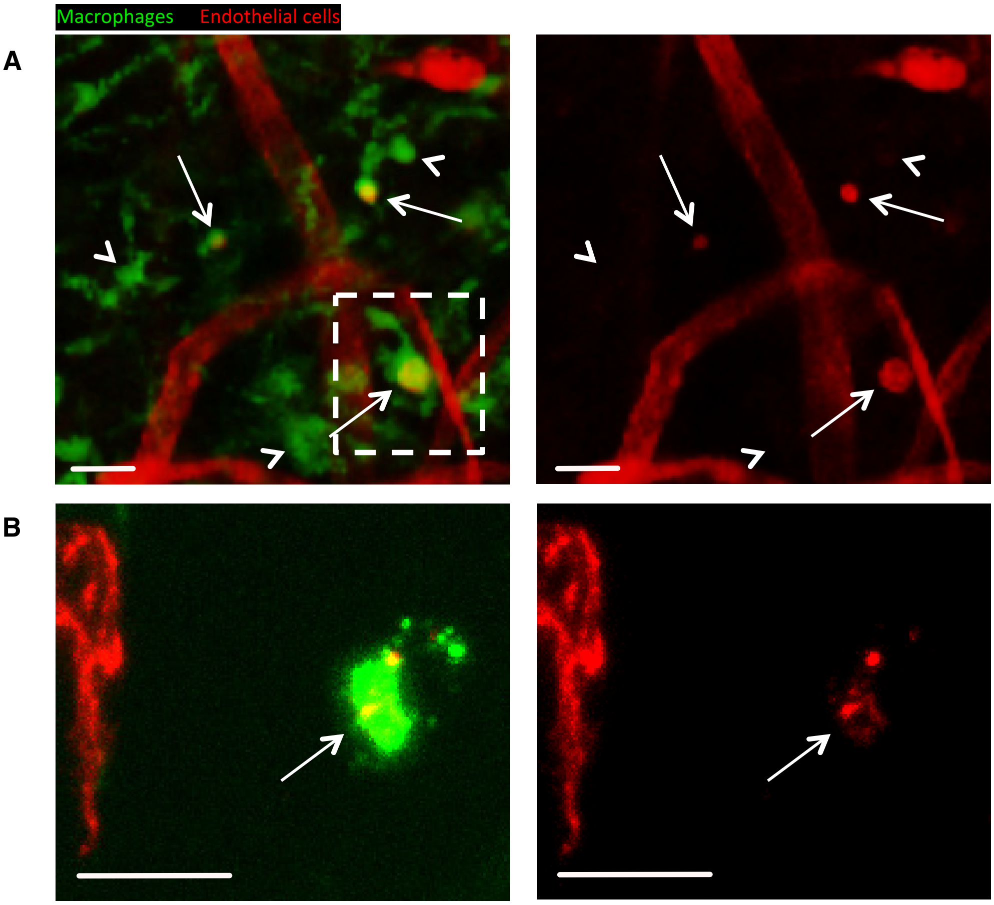

Figure EV1. Macrophage-mediated clearance of endothelial cells during blood vessel remodelling stages of healing mouse wounds.

A Expanded view of clearance stage image shown in Fig 1E (indicated by boxed area), demonstrating that some macrophages contain CD31-stained endothelial cell material (arrows), while others do not (arrowheads).

B Representative confocal projection images of frozen sections taken from 10 DPI mouse wounds stained for blood vessels (VE-Cadherin) and macrophages (CD68), which complement our CD31 stained whole mouse wounds, to further demonstrate endothelial cell matter within macrophages. *N* = 4 independent mice.

Data information: Scale bars, 20 µm.

Figure EV2. Dynamic vessel sprouting in response to tissue damage results in re-establishment of vessel patency within four days in zebrafish wounds.

A Representative fluorescent stereomicroscope images taken from 30-gauge needle-stick wounded Tg(*fli*:GFP) zebrafish. High molecular weight dextran was loaded one hour prior to injury, with images taken immediately after and one hour after injury, showing leakage of dextran (red) into the wound site upon vessel damage. *N* = 12 independent fish.

B Confocal projection images of a representative 30-gauge needle wounded Tg(*fli*:GFP) zebrafish, loaded with dextran one hour prior to imaging, showing the typical time course of blood vessel leakiness over 4 DPI following needle-stick injury as per (A). Vessels typically cease leaking dextran by 4 DPI. *N* = 12 independent fish per timepoint.

C Confocal projection images of a representative fine tungsten needle wounded Tg(*fli*:GFP) zebrafish, with damage induced to an existing vessel or between existing vessels. DIC images are provided as a guide to injury positioning. *N* = 8 independent fish per condition.

D Representative confocal projection images taken from timelapse movies of a laser injured Tg(*fli*:GFP); Tg(*mpx*:GFP) zebrafish, 4 DPF, imaged at 150–465 MPI, showing the dynamic nature of vessel tip extensions. *N* = 10 independent fish.

E Representative confocal projection images taken from timelapse movies of a laser injured Tg(*kdrl*:mCherry-CAAX); Tg(*mpx*:GFP); Tg(*mpeg*:mCherry) transgenic zebrafish, 4 DPF, imaged at 30–420 MPI (Movie EV5), to complement our dynamic studies of neutrophils and macrophages observed in Movie EV2. *N* = 5 independent fish.

Data information: Boxed area denotes wound site. Scale bars: (A) 100 µm; (B, C) 40 µm; (D, E) 20 µm.

© 2018 The Authors

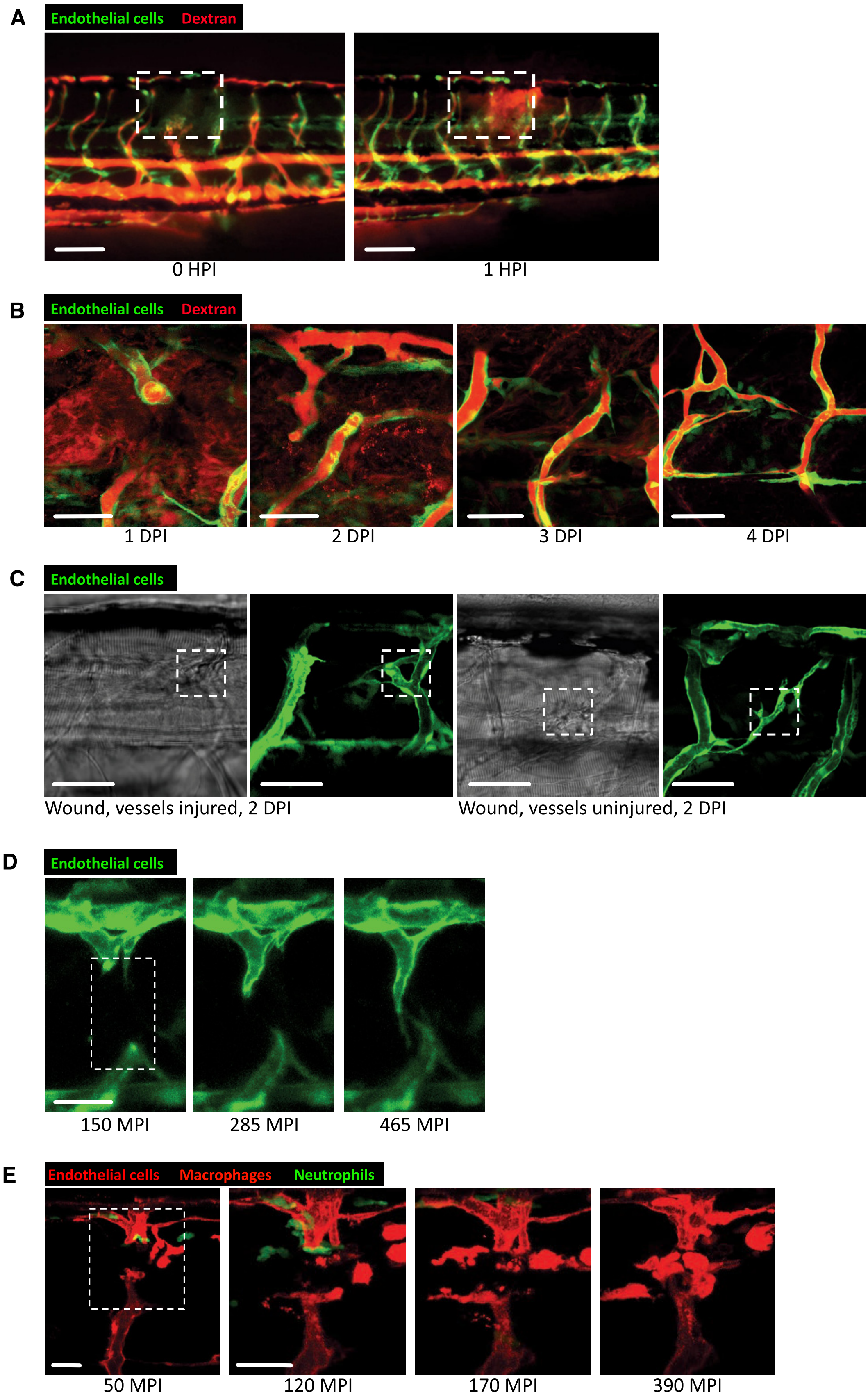
A
Endothelial cells Dextran
0 HPI
1 HPI
B
Endothelial cells Dextran
1 DPI
2 DPI
3 DPI
4 DPI
C
Endothelial cells
Wound, vessels injured, 2 DPI
Wound, vessels uninjured, 2 DPI
D
Endothelial cells
150 MPI
285 MPI
465 MPI
E
Endothelial cells Macrophages Neutrophils
50 MPI
120 MPI
170 MPI
390 MPI

Figure EV2.

Figure EV3. Extent of wound angiogenesis is proportional to number of pro-angiogenic macrophages versus anti-angiogenic neutrophils present at wound site.

A Representative fluorescent stereomicroscope images taken from Tg(*mpeg*:KalTA4); Tg(*UAS*:nfsB-mCherry) transgenic zebrafish, either treated with control DMSO or metronidazole as indicated. Metronidazole treatment resulted in > 90% macrophage ablation using this genetic model. N = 10 independent fish per condition.

B Representative fluorescent stereomicroscope images taken from Tg(*mpeg*:mCherry) transgenic zebrafish, injected with either control liposomes or clodronate liposomes, showing similar levels of macrophage ablation to metronidazole treatment. N = 10 independent fish per condition.

C Two metronidazole treatment protocols were used to ablate macrophages prior to injury, or throughout injury. Wounded Tg(*mpeg*:KalTA4); Tg(*UAS*:nfsB-mCherry); Tg (*fli*:GFP) transgenic zebrafish larvae were treated with DMSO (control) or metronidazole as indicated. Boxed area denotes wound site.

D Quantification of number of macrophages at the wound site of DMSO-treated control fish versus fish treated under the two ablation protocols, measured from images represented in (C) and Fig 2. Under protocol 1, fish recovered into fresh water post-injury saw a partial rescue of macrophage numbers at the wound site by 4 DPF. N = 12 independent fish per condition per timepoint.

E Quantification of total blood vessel length, measured from images represented in (C), using Angioanalyser. Under protocol 1, fish with partially rescued macrophages also showed a trend towards increased wound angiogenesis compared to full macrophage ablation. Treatment of Tg(*fli*:GFP) transgenic zebrafish with metronidazole showed no difference in extent of wound angiogenesis compared to untreated injury. N = 12 independent fish per condition per timepoint. Statistical significance, as determined by one-way ANOVA, is $P \leq 0.0001$. Subsequent Bonferonni multiple comparison test, determines level of significance, as indicated. Significance values: ***$P \leq 0.0001$.

F 1% agarose gel, showing that both mflt1 and sflt1 products are present in whole zebrafish cDNA, but only sflt1 cDNA is present in FAC-sorted Tg(*mpx*:GFP) neutrophils in early zebrafish wounds, as outlined in Fig 3F.

Data information: For all graphs, error bars indicate mean ± SD. Scale bars: (A, B) 200 μm; (C) 100 μm.

© 2018 The Authors

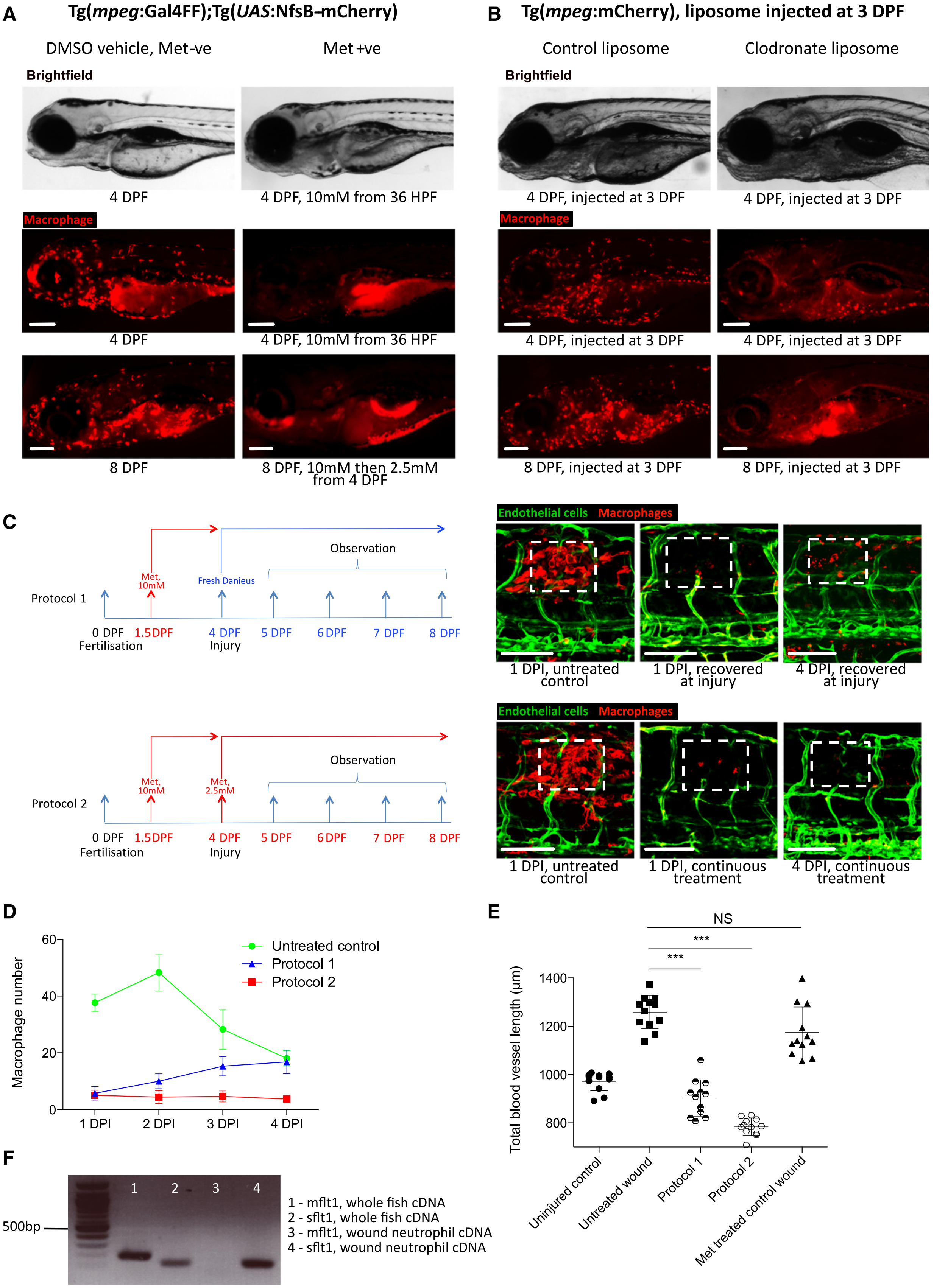
A
Tg(mpeg:Gal4FF);Tg(UAS:NfsB–mCherry)
DMSO vehicle, Met-ve
Met +ve
Brightfield
4 DPF
4 DPF, 10mM from 36 HPF
Macrophage
4 DPF
4 DPF, 10mM from 36 HPF
8 DPF
8 DPF, 10mM then 2.5mM from 4 DPF
B
Tg(mpeg:mCherry), liposome injected at 3 DPF
Control liposome
Clodronate liposome
Brightfield
4 DPF, injected at 3 DPF
4 DPF, injected at 3 DPF
Macrophage
4 DPF, injected at 3 DPF
4 DPF, injected at 3 DPF
8 DPF, injected at 3 DPF
8 DPF, injected at 3 DPF
C
Observation
Met, 10mM
Fresh Danieus
Protocol 1
0 DPF Fertilisation
1.5DPF
4 DPF Injury
5 DPF
6 DPF
7 DPF
8 DPF
Endothelial cells Macrophages
1 DPI, untreated control
1 DPI, recovered at injury
4 DPI, recovered at injury
Observation
Met, 10mM
Met, 2.5mM
Protocol 2
0 DPF Fertilisation
1.5DPF
4 DPF Injury
5 DPF
6 DPF
7 DPF
8 DPF
Endothelial cells Macrophages
1 DPI, untreated control
1 DPI, continuous treatment
4 DPI, continuous treatment
D
Macrophage number
60
40
20
0
1 DPI
2 DPI
3 DPI
4 DPI
Untreated control
Protocol 1
Protocol 2
E
NS
***
***
Total blood vessel length (µm)
1400
1200
1000
800
Uninjured control
Untreated wound
Protocol 1
Protocol 2
Met treated control wound
F
500bp
1
2
3
4
1 - mflt1, whole fish cDNA
2 - sflt1, whole fish cDNA
3 - mflt1, wound neutrophil cDNA
4 - sflt1, wound neutrophil cDNA

Figure EV3.

Figure EV4. Manipulation of normally dynamic macrophage phenotype towards a pro-inflammatory state alters the extent of wound angiogenesis and the patency of vessels.

A Representative confocal projection images taken from either 1 DPI Tg(*mpeg*:mCherry); Tg(*tnfα*:GFP) transgenic zebrafish (top panel) or 4 DPI Tg(*fli*:GFP) transgenic fish (bottom panel), wild type or *csf1ra*$^{-/-}$ mutant, treated as indicated with LPS or hydrocortisone. Boxed area denotes wound site.

B Quantification of number of macrophages at the wound site from 1 to 4 DPI, measured from images represented in (A) and Fig 4. $N = 14$ independent fish per timepoint per condition.

C Quantification of proportion of macrophages that are *tnfα* positive at the wound site from 1 to 4 DPI, measured from images represented in (A) and Fig 4. $N = 14$ independent fish per timepoint per condition.

D Quantification of total blood vessel length at 4 DPI, measured using Angioanalyser from images represented in (A). $N = 14$ independent fish per timepoint per condition. Statistical significance, as determined by one-way ANOVA, is $P \leq 0.0001$. Subsequent Bonferroni multiple comparison test, determines level of significance, as indicated. Significance values: $*P \leq 0.05$, $**P \leq 0.001$.

E Representative confocal projection images of 30-gauge needle wounded Tg(*fli*:GFP) zebrafish larvae, treated with DMSO, LPS or Ifn-γ from moment of injury, loaded with dextran (red) one hour prior to imaging, imaged at 4 DPI. Boxed area indicates wound site.

F Representative confocal projection images taken from laser wounded Tg(*kdrl*:mCherry-CAAX); Tg(*mpeg*:mCherry); Tg(*tnfα*:GFP) transgenic zebrafish, imaged 30–480 MPI. Similar early recruitment was observed with respect to *tnfα*-positive macrophages (arrowheads) versus *tnfα*-negative macrophages (asterisks), complementing our results from Movie EV8. As time progresses, some *tnfα*-positive macrophages diminish their GFP expression as they leave the site of injury (arrow). Boxed area denotes wound site. $N = 5$ independent fish.

Data information: For all graphs, error bars indicate mean ± SD. Scale bars: (A, E) 100 μm; (F) 40 μm.

© 2018 The Authors

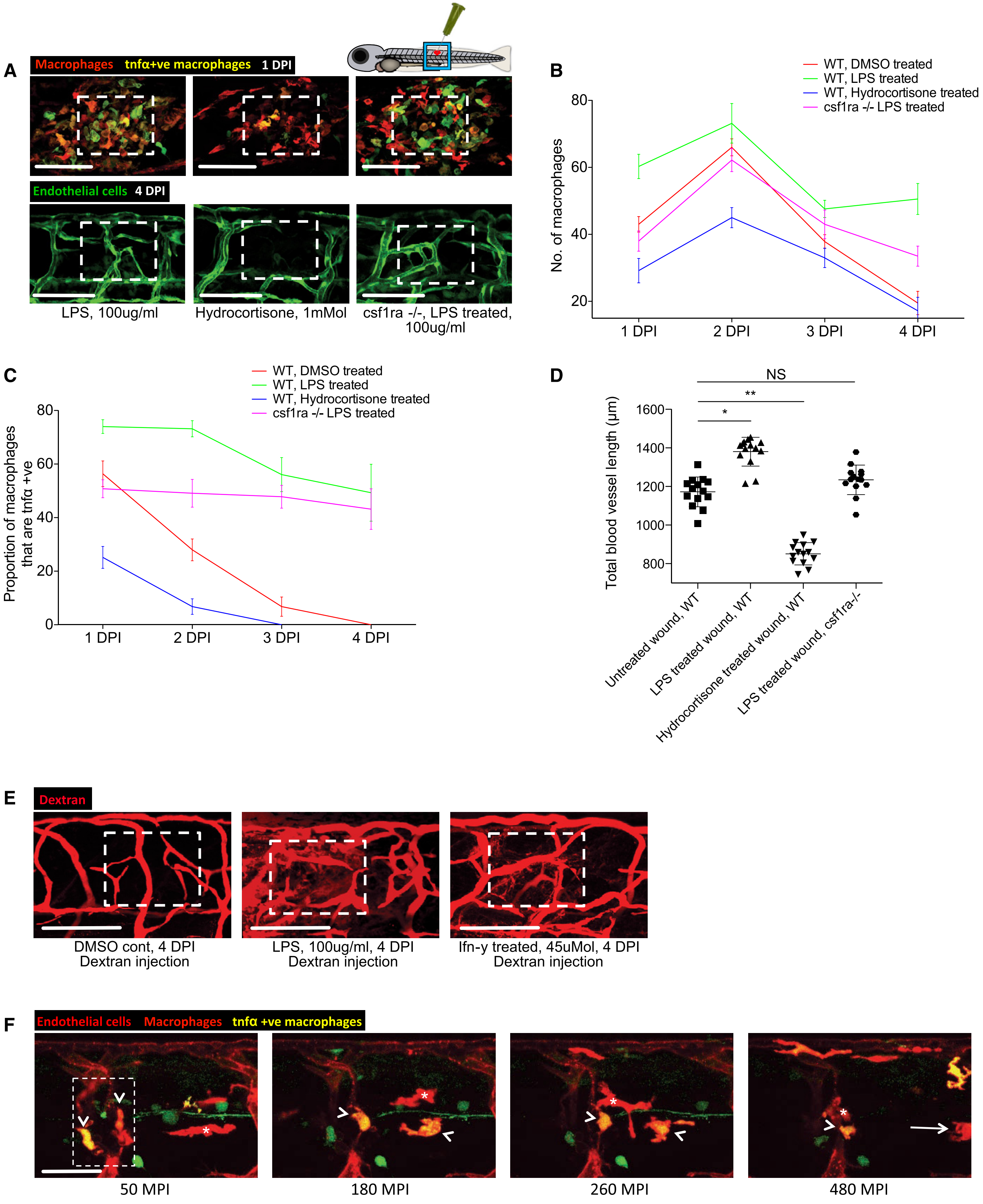
A
Macrophages tnfα+ve macrophages 1 DPI
Endothelial cells 4 DPI
LPS, 100ug/ml
Hydrocortisone, 1mMol
csf1ra -/-, LPS treated, 100ug/ml
B
WT, DMSO treated
WT, LPS treated
WT, Hydrocortisone treated
csf1ra -/- LPS treated
No. of macrophages
80
60
40
20
1 DPI
2 DPI
3 DPI
4 DPI
C
WT, DMSO treated
WT, LPS treated
WT, Hydrocortisone treated
csf1ra -/- LPS treated
Proportion of macrophages that are tnfα +ve
80
60
40
20
0
1 DPI
2 DPI
3 DPI
4 DPI
D
NS
**
*
Total blood vessel length (μm)
1600
1400
1200
1000
800
Untreated wound, WT
LPS treated wound, WT
Hydrocortisone treated wound, WT
LPS treated wound, csf1ra-/-
E
Dextran
DMSO cont, 4 DPI
Dextran injection
LPS, 100ug/ml, 4 DPI
Dextran injection
Ifn-y treated, 45uMol, 4 DPI
Dextran injection
F
Endothelial cells Macrophages tnfα +ve macrophages
50 MPI
180 MPI
260 MPI
480 MPI

Figure EV4.

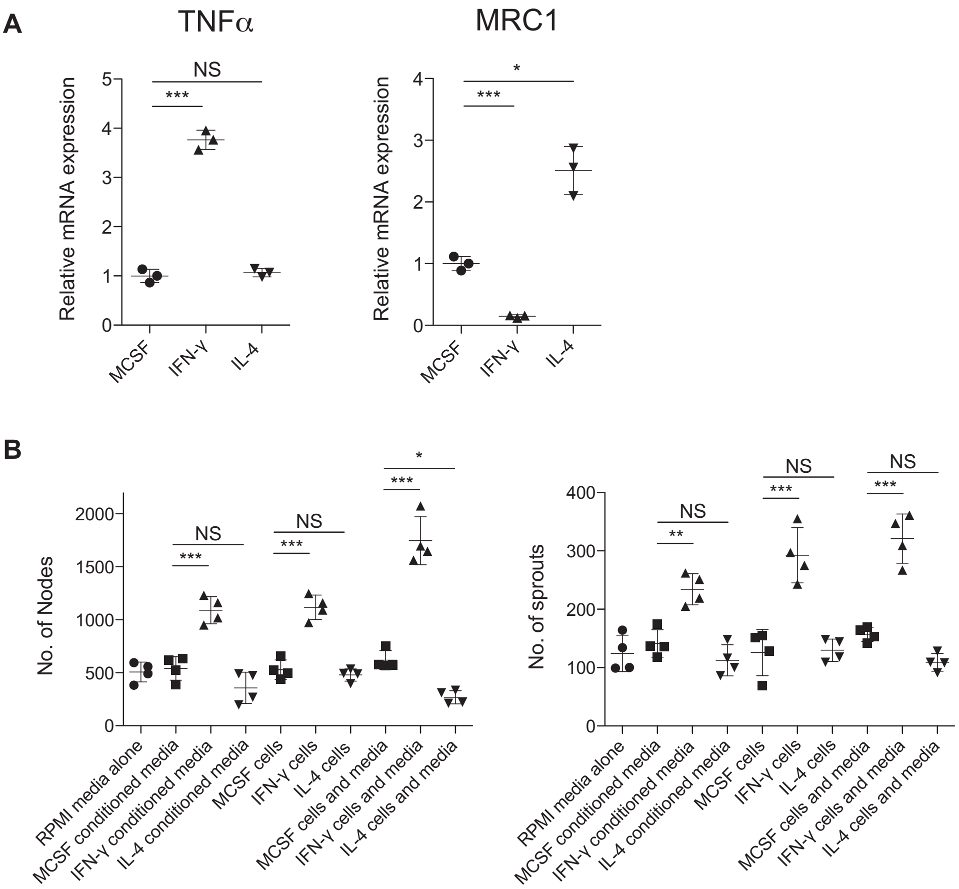

Figure EV5. Inflammatory macrophages increase both extent and complexity of the resultant blood vessels in human HUVEC/macrophage co-culture.

A TNFα and MRC1 expression levels for human macrophages treated with IFN-γ versus IL-4, compared to control macrophages treated with MSCF alone. $N = 3$ independent macrophage culture experiments per condition. Statistical significance, as determined by one-way ANOVA, is $P \leq 0.0001$.

B Quantification of vessel complexity (nodes and sprouts, as indicated) of HUVECs cultured or co-cultured as indicated, measured from images represented in Fig 5D, using Angioanalyser. Statistical significance, as determined by one-way ANOVA, is $P \leq 0.0001$.

Data information: Subsequent Bonferroni multiple comparison test, determines level of significance, as indicated. Significance values: $^{*}P \leq 0.05$, $^{**}P \leq 0.001$, $^{***}P \leq 0.0001$. For all graphs, error bars indicate mean ± SD.

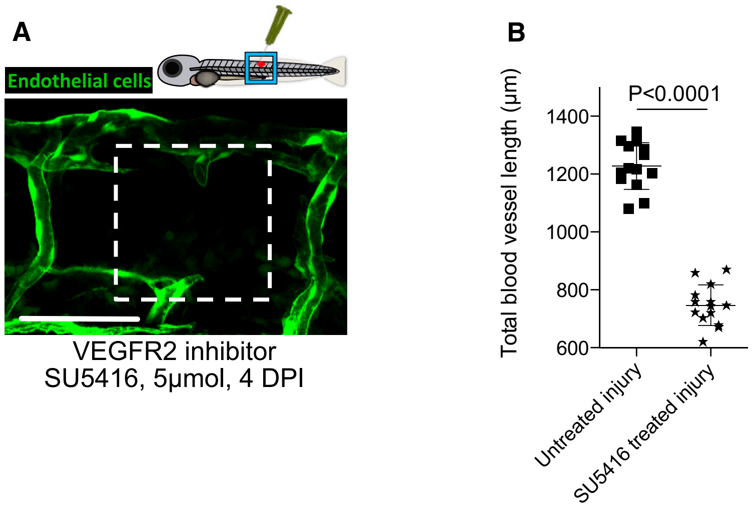

Figure EV6. Suppression of vegf signalling causes failure of wound angiogenesis in zebrafish.

A Representative confocal projection image taken from needle-stick wounded Tg(*fli*:GFP) transgenic zebrafish, imaged 4 DPI and treated with vegfr2 inhibitor SU5416 from the moment of injury. Boxed area denotes wound site. Scale bar, 100 µm.

B Quantification of total blood vessel length, measured from images represented in (A) using Angioanalyser. $N = 10$ independent fish. Statistical significance is indicated, as determined by two-tailed *t*-test. Error bars indicate mean ± SD.

© 2018 The Authors
