# Supplementary material for: Live imaging of wound angiogenesis reveals macrophage orchestrated vessel sprouting and regression
Source: EMBO J. 2018 Jun 4;37(13):e97786. doi: 10.15252/embj.201797786 (PMC6028026; doi:10.15252/embj.201797786)
Supplement: Supplementary file 2 — Movie EV1 [file EMBJ-37-e97786-s002.zip › Movie_1_legend.docx]

**Movie 1** – Slice by slice z-stack movie of the antibody stained macrophage pictured in the ‘Clearance’ image of Figure 1E, showing endothelial cell matter in a discrete bolus within the macrophage.
