# Supplementary material for: Live imaging of wound angiogenesis reveals macrophage orchestrated vessel sprouting and regression
Source: EMBO J. 2018 Jun 4;37(13):e97786. doi: 10.15252/embj.201797786 (PMC6028026; doi:10.15252/embj.201797786)
Supplement: Supplementary file 3 — Movie EV2 [file EMBJ-37-e97786-s003.zip › Movie_2_legend.docx]

**Movie 2 –** Representative timelapse movie of a laser wounded, partial vessel ablated Tg(*fli*:GFP); Tg(*mpx*:GFP); Tg(*mpeg*:mCherry) transgenic zebrafish, 4 DPF, imaged every 15 minutes, 30-930 MPI.
