# Supplementary material for: Live imaging of wound angiogenesis reveals macrophage orchestrated vessel sprouting and regression
Source: EMBO J. 2018 Jun 4;37(13):e97786. doi: 10.15252/embj.201797786 (PMC6028026; doi:10.15252/embj.201797786)
Supplement: Supplementary file 4 — Movie EV3 [file EMBJ-37-e97786-s004.zip › Movie_3_legend.docx]

**Movie 3 -** Representative timelapse movie of a laser wounded, full vessel ablated Tg(*fli*:GFP); Tg(*mpx*:GFP) transgenic, showing dynamic filopodial extensions following full ISV ablation, 4 DPF, imaged every 15 minutes, 150-465 MPI.
