# Supplementary material for: Live imaging of wound angiogenesis reveals macrophage orchestrated vessel sprouting and regression
Source: EMBO J. 2018 Jun 4;37(13):e97786. doi: 10.15252/embj.201797786 (PMC6028026; doi:10.15252/embj.201797786)
Supplement: Supplementary file 5 — Movie EV4 [file EMBJ-37-e97786-s005.zip › Movie_4_legend.docx]

**Movie 4 –** 3D projection of repaired vessel at the final timepoint of Movie 2, showing continued contact between vessel and macrophages.
