# Supplementary material for: Live imaging of wound angiogenesis reveals macrophage orchestrated vessel sprouting and regression
Source: EMBO J. 2018 Jun 4;37(13):e97786. doi: 10.15252/embj.201797786 (PMC6028026; doi:10.15252/embj.201797786)
Supplement: Supplementary file 6 — Movie EV5 [file EMBJ-37-e97786-s006.zip › Movie_5_legend.docx]

**Movie 5 –** Representative timelapse movie of a laser wounded, partial vessel ablated Tg(*kdrl*:mCherry-CAAX); Tg(*mpx*:GFP); Tg(*mpeg*:mCherry) transgenic zebrafish, 4 DPF, imaged every 10 minutes, 30-420 MPI.
