# Supplementary material for: Live imaging of wound angiogenesis reveals macrophage orchestrated vessel sprouting and regression
Source: EMBO J. 2018 Jun 4;37(13):e97786. doi: 10.15252/embj.201797786 (PMC6028026; doi:10.15252/embj.201797786)
Supplement: Supplementary file 7 — Movie EV6 [file EMBJ-37-e97786-s007.zip › Movie_6_legend.docx]

**Movie 6 -** Representative timelapse movie of laser wounded, full vessel ablated Tg(*fli*:GFP); Tg(*mpx*:GFP); Tg(*mpeg*:mCherry), treated with 2.5mMol metronidazole (treatment control), 4 DPF, imaged every 20 minutes, 30-930 MPI.
