# Supplementary material for: Live imaging of wound angiogenesis reveals macrophage orchestrated vessel sprouting and regression
Source: EMBO J. 2018 Jun 4;37(13):e97786. doi: 10.15252/embj.201797786 (PMC6028026; doi:10.15252/embj.201797786)
Supplement: Supplementary file 8 — Movie EV7 [file EMBJ-37-e97786-s008.zip › Movie_7_legend.docx]

**Movie 7 -** Representative timelapse movie of laser wounded, full vessel ablated Tg(*fli*:GFP); Tg(*mpx*:GFP); Tg(*mpeg*:KalTA4); Tg(*UAS*:nfsB-mCherry) macrophage ablation transgenic, treated with 2.5mMol metronidazole (treatment control), 4DPF, imaged every 20 minutes, 30-930 MPI.
