# Supplementary material for: Live imaging of wound angiogenesis reveals macrophage orchestrated vessel sprouting and regression
Source: EMBO J. 2018 Jun 4;37(13):e97786. doi: 10.15252/embj.201797786 (PMC6028026; doi:10.15252/embj.201797786)
Supplement: Supplementary file 9 — Movie EV8 [file EMBJ-37-e97786-s009.zip › Movie_8_legend.docx]

**Movie 8 -** Representative timelapse movie of laser wounded, vessel ablated Tg(*fli*:GFP); Tg(*mpeg*:mCherry); Tg(*tnfα*:GFP) transgenic zebrafish, 4 DPF, imaged every 15 minutes, 30-930 MPI.
